# Editorial: Learning and skill acquisition in sports: theoretical perspectives

**DOI:** 10.3389/fspor.2023.1360500

**Published:** 2024-01-15

**Authors:** Henrik Herrebrøden, Rob Gray, Thomas Schack, Christian Thue Bjørndal

**Affiliations:** ^1^ Department of Psychology, Pedagogy and Law, School of Health Sciences, Kristiania University College, Oslo, Norway; ^2^ Human Systems Engineering, Arizona State University, Mesa, AZ, United States; ^3^ Faculty of Psychology and Sports Science, Neurocognition and Action-Biomechanics-Research Group, Bielefeld University, Bielefeld, Germany; ^4^ Department of Sport and Social Sciences, Norwegian School of Sport Sciences, Oslo, Norway; ^5^ Norwegian Research Centre for Children and Youth Sports, Norwegian School of Sport Sciences, Oslo, Norway

**Keywords:** learning, skill acquisition, sports, information processing, ecological dynamics, theory

**Editorial on the Research Topic** Learning and Skill Acquisition in Sports: Theoretical Perspectives

## Introduction

The sports literature offers a diverse selection of theoretical views that attempt to explain how athletes improve with practice. This topic has significant implications for coaches, practitioners, and athletes themselves. Faced with the complexity of this subject, a common tendency is to simplify the issue by categorizing within a dichotomy (1), as evidenced by the common distinction between frameworks based on mental models or representations (e.g., information processing), on the one hand, and ecological approaches (e.g., ecological dynamics), on the other. While the former theoretical frameworks emphasize cognitive factors as keys to performance and improvement, the latter emphasize the relationship between the individual and the environment. The debate between these opposing perspectives has dominated discussions in sport psychology and movement science for over four decades. As suggested by Ranganathan and Driska in the present Research Topic and reinforced by the review conducted by Ashford et al. (2), both the information-processing and ecological-dynamics perspectives tend to generate data, terminology, and interpretations aligned with their respective frameworks.

Despite these binary perspectives and ongoing debates, the validity of the proposed dichotomy is not entirely clear, and how these views differ from or complement each other remains unsettled (2, 3). Moreover, several other theoretical perspectives on learning and skill acquisition in sports may not neatly align with the dichotomy between cognitive models and ecological frameworks.

These issues inspired the current Research Topic, in which scholars were invited to shed light on various approaches to skill acquisition and learning in sports. In the following, we attempt to summarize and offer some conclusions based on the various contributions.

### Theoretical comparisons and commentaries

Koester addressed the ongoing debate between dynamic systems theory (DST) and symbol processing accounts (SPA), highlighting the limitations of both approaches not only from a theoretical standpoint but also in terms of practical consequences for skill learning. He argued for a more comprehensive perspective to support skill learning and rehabilitation, advocating an action-centered perspective and a cognitive future of motor control and learning. Gottwald et al. evaluated the information-processing and ecological-dynamics approaches in the context of focus of attention in skill acquisition. The authors provided a detailed review of relevant research and asserted that the functionality of an appropriate focus of attention is predominantly addressed from an information-processing perspective, with less emphasis from an ecological standpoint. To address this, the authors suggested a novel and more flexible perspective called “Ecological Dynamics Account of Attentional Focus,” accompanied with practical recommendations. In their contribution, Ranganathan and Driska raised the question of whether premature theorizing negatively influences skill acquisition research. Discussing the ongoing debate between the information-processing and ecological-dynamics approaches, the authors contended that the limited data on skill acquisition research impedes the conclusive determination of the quality or utility of these respective theories. They went on to provide recommendations for the research field, from both a researcher's and a practitioner's perspective.

### Ecological approaches

A prevalent theme across many articles is the application of the ecological-dynamics framework to skill acquisition, from specific sporting examples to a broader consideration of practice design. For example, Ziv illustrated how racecar driving can be understood by considering the driver, the car, and the racecourse as an interconnected system. From  this perspective, skill is considered in terms of the constraints faced by the driver and the perception of affordances (such as passing, braking). O'Sullivan et al. discussed the application of an ecological framework in a case study of a youth football club. In particular, they highlighted the importance of considering sociocultural constraints in player development. Similarly, Rothwell et al., in their case study of a wheelchair rugby team, illustrated how the transfer of knowledge between coach and player can be understood from an ecological perspective as a bidirectional self-organizing system. Addressing the general issue of skill acquisition, Myszka et al. presented a conceptualization of skill acquisition as a problem-solving activity, where performers strive to find the best movement solution to achieve their goal under ever-changing constraints. Finally, Chow et al. offered reflections on how the ecological-dynamics theory can be applied in coaching and practice design. The  authors addressed some common concerns including the overusing of jargon, quantifying improvements over time, and giving up control as a coach.

### Beyond the dichotomy

The diversity of philosophical and theoretical perspectives significantly contributes to the advancement of research on skilled movement in sports, moving beyond information processing and ecological dynamics. Engelsrud's work served as an example, showcasing how a phenomenology-informed approach can explore movement experience in yoga practice, emphasizing bodily resonances and sensuous interactions among individuals. Similarly, the study of Stien et al. on non-verbal, visual feedback in resistance training demonstrated the application of this approach to traditional skill adaptation research and practice design. Notably, this novel method blurs and eliminates conventional distinctions between internal and external stimuli and feedback, especially when considering alternative experimental setups and conceptualizations. While there is an ontological resemblance to ecological psychology, it is crucial to acknowledge the divergence in epistemic traditions. Future research should explore how these perspectives can mutually inform theory, research, and practice.

The significance of understanding an athlete's past development and its influence on future learning and skill development in sports was evidenced by the work of Papastaikoudis et al. Through their examination of childhood experiences and psychological skills among youth athletes, they underscored the significance of considering past experiences and psychological resources when designing appropriate learning environments and practice designs. Taking this idea further, Rossing et al. proposed a conceptualization of learning through sports that extends beyond the traditional notion of movement learning. They perceived sports participation as a situated and social practice, where diverse meanings are experienced and negotiated, significantly influencing the lives of young individuals. Drawing  from their case study within disability sports, they present examples that could compel movement scientists to be highly attentive to the social context's influence on learning and skill acquisition. This extends beyond the acquisition and adaptation of sport-specific skills, prompting a reconsideration of our preconceptions about what constitutes appropriate session designs.

Finally, adopting a macroscopic lens, Herrebrøden and Bjørndal explored the (in)significance of youth international experience for senior success in football across six European countries and various playing positions (see also their corrigendum).

## Conclusion

The current contributions offer several takeaway messages. Notably, empirical studies can be guided and interpreted through the lens of different theoretical frameworks. This was most often exemplified by ecological approaches, which can be applied to explain a variety of findings and phenomena, including ones that have traditionally been associated with information-processing views, such as attentional focus effects and knowledge transfer. However, some theoretical approaches do not readily fit into the dichotomy of processing-based vs. ecological approaches. The field of skill acquisition in sports is still in its infancy, and more work will aid the evaluation of theoretical frameworks. In light of the current Research Topic, we hope that the future literature will see diverse contributions, both empirical and theoretical, addressing various aspects related to learning in sport contexts.
